# Aerobic glycolysis imaging of epileptic foci during the inter-ictal period

**DOI:** 10.1016/j.ebiom.2022.104004

**Published:** 2022-04-15

**Authors:** Miao Zhang, Qikai Qin, Shuning Zhang, Wei Liu, Hongping Meng, Mengyang Xu, Xinyun Huang, Xiaozhu Lin, Mu Lin, Peter Herman, Fahmeed Hyder, Raymond C. Stevens, Zheng Wang, Biao Li, Garth J. Thompson

**Affiliations:** a Department of Nuclear Medicine, Ruijin Hospital, Shanghai Jiao Tong University School of Medicine, Shanghai 200025, China; b iHuman Institute, ShanghaiTech University, Shanghai 201210, China; c School of Life Science and Technology, ShanghaiTech University, Shanghai 201210, China; d University of Chinese Academy of Sciences, Beijing 100049, China; e Department of Neurosurgery, Ruijin Hospital, Shanghai Jiao Tong University School of Medicine, Shanghai 200025, China; f Shanghai Institute of Biochemistry and Cell Biology, Center for Excellence in Molecular Cell Science, Chinese Academy of Sciences, Shanghai 200031, China; g MR Collaboration, Siemens Healthineers Ltd., Shanghai 201318, China; h Magnetic Resonance Research Center (MRRC), Yale University, New Haven 06520, USA; i Quantitative Neuroscience with Magnetic Resonance (QNMR) Core Center, Yale University, New Haven 06520, USA; j Radiology and Biomedical Imaging, Yale University, New Haven 06520, USA; k Biomedical Engineering, Yale University, New Haven 06520, USA; l School of Psychological and Cognitive Sciences; Beijing Key Laboratory of Behavior and Mental Health; IDG/McGovern Institute for Brain Research; Peking-Tsinghua Center for Life Sciences, Peking University, Beijing 100871, China; m Collaborative Innovation Center for Molecular Imaging of Precision Medicine, Ruijin Center, Shanghai 200025, China

**Keywords:** Oxygen-glucose index, Epilepsy, Inter-ictal, Calibrated fMRI, Lactate, Biomarker

## Abstract

**Background:**

In drug-resistant epilepsy, surgical resection of the epileptic focus can end seizures. However, success is dependent on the ability to identify foci locations and, unfortunately, current methods like electrophysiology and positron emission tomography can give contradictory results. During seizures, glucose is metabolized at epileptic foci through aerobic glycolysis, which can be imaged through the oxygen-glucose index (OGI) biomarker. However, inter-ictal (between seizures) OGI changes have not been studied, which has limited its application.

**Methods:**

18 healthy controls and 24 inter-ictal, temporal lobe epilepsy patients underwent simultaneous positron emission tomography (PET) and magnetic resonance imaging (MRI) scans. We used [^18^F]fluorodeoxyglucose-PET (FDG-PET) to detect cerebral glucose metabolism, and calibrated functional MRI to acquire relative oxygen consumption. With these data, we calculated relative OGI maps.

**Findings:**

While bilaterally symmetrical in healthy controls, we observed, in patients during the inter-ictal period, higher OGI ipsilateral to the epileptic focus than contralateral. While traditional FDG-PET results and temporal lobe OGI results usually both agreed with invasive electrophysiology, in cases where FDG-PET disagreed with electrophysiology, temporal lobe OGI agreed with electrophysiology, and vice-versa.

**Interpretation:**

As either our novel epilepsy biomarker or traditional approaches located foci in every case, our work provides promising insights into metabolic changes in epilepsy. Our method allows single-session OGI measurement which can be useful in other diseases.

**Funding:**

This work was supported by ShanghaiTech University, the Shanghai Municipal Government, the National Natural Science Foundation of China Grant (No. 81950410637) and Shanghai Municipal Key Clinical Specialty (No. shslczdzk03403). F. H. and P. H. were supported by USA National Institute of Health grants (R01 NS-100106, R01 MH-067528).Z. W. was supported by the Key-Area Research and Development Program of Guangdong Province (2019B030335001), National Natural Science Foundation of China (No. 82151303), and National Key R&D Program of China (No. 2021ZD0204002).

**Research in Context**

**Evidence before this study**

In epilepsy, patients’ quality of life is dramatically reduced by seizures, caused by brain activity emerging from some part of the brain. Which part of the brain causes seizures will differ between patients, but these brain regions are collectively referred to as epileptic foci. In patients where drugs are ineffective, surgery to remove the epileptic focus may help, but it must be located first. Invasive electrical recording can locate foci but, prior to this surgery, the approximate location must be found via non-invasive imaging. However, non-invasive imaging often disagrees with invasive electrical recording, and the cost of this mistake is high. Thus, further non-invasive methods are urgently needed. One such method may come from an understanding of metabolism. The first step in metabolizing glucose, glycolysis, doesn't require oxygen, so will relatively increase if there is a lack of oxygen. But, when a larger than normal amount of glycolysis occurs despite sufficient oxygen being present, this is known as “aerobic glycolysis.” Epileptic foci undergo increased aerobic glycolysis during seizures, and likely their aerobic glycolysis changes between seizures as well, though the latter has not been studied yet. Thus far, studies have been limited as current methods require multiple imaging sessions or rarely available, radioactive chemicals. However, combined PET-MRI systems allow us to measure oxygen with MRI, and glucose with PET, and thus non-invasively in a single session measure the OGI, a measure of aerobic glycolysis.

**Added value of this study**

We measured aerobic glycolysis using OGI in both healthy controls and epilepsy patients in a single session using the combined PET-MRI method. We chose patients who had epilepsy located in their temporal lobes, and imaged their brains in the period between seizures. While healthy controls had the same OGI on both sides of their temporal lobes, patients had significantly higher OGI on the side of their epileptic foci than the other side, indicating less aerobic glycolysis. While in most cases both the current standard method and our novel method agreed with invasive electrical recording, in all cases where the standard method disagreed with invasive electrical recording, our method agreed with it.

**Implications of all the available evidence**

Our work demonstrates that in temporal lobe epilepsy patients in the period between seizures, aerobic glycolysis is lower near the epileptic focus than the other side. Furthermore, aerobic glycolysis provided additional diagnostic information as it agreed with invasive electrical recording whenever standard methods did not. Beyond epilepsy, our combined PET-MRI method for OGI also could be applied to other diseases where aerobic glycolysis is disturbed, such as effects of previous malnutrition, Huntington's disease, Alzheimer's disease, and metabolic disorders. Our method is easily implemented in combined PET-MRI scanners which have already been installed in many hospitals worldwide, with the number expected to increase in the near future.

Alt-text: Unlabelled box

## Introduction

Affecting about 65 million people worldwide,^1^ epilepsy is a chronic and recurrent disorder of the brain characterized by epileptic seizures due to abnormally excessive or synchronous neuronal activity, usually emerging from a brain region known as the epileptic focus. In drug-resistant epilepsy, resecting the epileptic focus can end seizures, but it must be located first. For magnetic resonance imaging (MRI) negative epilepsy, one currently-used method first requires a positron emission tomography (PET) of [^18^F]fluorodeoxyglucose (FDG) to locate a brain region with reduced glucose metabolism, followed by the surgical implantation of electrodes to record a stereo-electroencephalograph (SEEG) signal to precisely locate the epileptic focus prior to resection. However, FDG-PET results can disagree with SEEG,^2^ and since SEEG and seizure resection are invasive, the cost of misdiagnosis is high. Thus, non-invasive approaches to characterizing epileptic foci would significantly benefit disease treatment.

One characteristic of epileptic foci, not used in current evaluation criteria, is that they undergo “aerobic glycolysis” during seizures. Here, even though oxygen is present in sufficient amounts for glucose to be fully oxidized, glycolysis dominates to shunt pyruvate towards lactate instead of acetyl CoA. The oxygen-glucose index (OGI) is defined as the ratio of oxygen consumption (CMR_O2_) to glucose consumption (CMR_glc_), and thus the inverse of OGI in the brain reflects the extent of aerobic glycolysis.^3^^,^^4^ OGI has already been used as a neurological biomarker in studies of malnutrition recovery^5^ and Huntington's Disease.^6^ Furthermore, even healthy subjects show a modest decrease in OGI during normal brain activity.^7^ Since aerobic glycolysis is known to occur at epileptic foci during seizures when they both accumulate and release larger than normal amounts of lactate,^8^ OGI is a potential method for both locating epileptic foci and also diagnosing epilepsy based on metabolic characteristics.

However, two key issues make measuring OGI in patients with epilepsy difficult. First, while existing literature only describes OGI changes during seizures,^8^ scanning during seizures presents major problems, including difficulties with scheduling scans to coincide with seizures, as well as the heightened risk to patients who might be experiencing the convulsions of *grand mal* seizures and might be unable to communicate. Therefore, it is much preferable to undertake clinical imaging of patients with epilepsy when they are between seizures, i.e., during the “inter-ictal” period. However, the effect of epilepsy on OGI during this inter-ictal period is unknown.

Second, PET is the classical method of measuring OGI, but this approach presents major problems in clinical imaging. Here, the independent measurement of oxygen metabolism and glucose metabolism requires two separate tracers, FDG for glucose and ^15^O-O_2_ for oxygen. ^15^O-O_2_ is not widely available and it also has a dramatically shorter half-life (∼2 minutes) than FDG (∼2 hours). Therefore, measuring both in the same session requires many technical assumptions that complicate OGI calculations. Therefore, past OGI measurements (in healthy subjects) have typically involved collecting FDG and ^15^O-O_2_ scans on different days,^3^^,^^4^ complicating clinical use.

Here, we present a study of OGI in epilepsy patients and healthy controls that focused on changes in OGI at patients’ epileptic foci using a single measurement session. It is well-established that, at epileptic foci, glucose metabolism is lower during the inter-ictal period relative to both surrounding tissue and the contralateral side.^1^^,^^9^ However, changes in glucose can be difficult to interpret without also knowing changes in oxygen metabolism. OGI could reveal aerobic glycolysis changes which are due to oxygen and glucose consumption changing together when either individual change could be too small to detect.

To perform OGI imaging in a single session using a combined PET-MRI (magnetic resonance imaging) scanner, we developed a technique that measures oxygen metabolism with a calibrated functional MRI (fMRI) method instead of PET^10^ methods, which require multiple scanning sessions,^3^ increasing patient stress, number of hospital visits, and radiation exposure. Rather than the blood-oxygen level dependent (BOLD) signal measured by traditional fMRI, calibrated fMRI methods measure CMR_O2_ through a biophysical model for BOLD signal. While many calibrated fMRI methods require administering CO_2_, we used a method that was recently developed in rodents^11^ that uses relaxometry instead of gas exposure.^10^^,^^12^, ^13^, ^14^ As modern PET-MRI scanners allow simultaneous recording of both modalities, calibrated fMRI can be combined with FDG-PET to measure both oxygen and glucose and achieve OGI measurement in a single session. Our approach allows the detection of altered OGI at epileptic foci versus the contralateral brain location, providing a new diagnostic parameter in terms of locating the lateralization of the epileptic focus and a better understanding of the metabolic effects of epilepsy.

## Methods

### Subjects

In this study, 24 patients (37.5% female, 30.7±9.7 years old) with intractable medial temporal lobe epilepsy, and 18 controls (44.4% female, 48.1±9.6 years old) were enrolled to compare between brain hemispheres using PET-MRI measurements of physiology, including the biomarker OGI. Each subject provided one measurement. All participants were recruited and scanned by Ruijin Hospital from August 2018 to July 2020. All patients underwent surgical resection after the SEEG evaluation and a minimum of 12 months postoperative follow-up to assess seizure outcome using the Engel Epilepsy Surgery Outcome Scale.^15^ Seizure outcomes of patients were rated from Class I to Class IV. The mean follow-up period was 15.5±5.6 months (range 12∼30 months). Twenty-one patients who met the criteria for Engel class I were classified as seizure-free. Three patients who met the criteria for Engel Class II or III (one or more recurrent complex partial or secondarily generalized seizures) were classified as not seizure-free (Table 1).

**Table 1:** Demographic data of healthy controls and epilepsy patients.

Clinical characteristics	Healthy control	Temporal lobe epilepsy
Number of subjects, n	18	24
Gender, Female, n (%)	8 (44.4%)	9 (37.5%)
Age at evaluation (years), mean ± SD (range)	48.1±9.6 (36-63)	30.7±9.9 (10-46)
Age of onset (years), mean ± SD (range)	/	16.6±10.4 (2-38)
Course of epilepsy (years), mean ± SD (range)	/	14.1±9.9 (1-40)
Seizure frequency (per year), mean ± SD (range)	/	41.7 ± 38.1 (4-120)
Left epilepsy, n (%)	/	18 (75.0%)
Postsurgical outcome, n (%)		
Engel Class Ⅰ	/	21 (87.5%)
Engel Class Ⅱ-Ⅳ	/	3 (12.5%)
Follow up duration, month, mean ± SD (range)	/	15.5±5.7 (12-30)

### Inclusion and exclusion criteria

For the healthy control group, the inclusion criteria were: (1) the subject went through an overall physical exam; (2) the subject does not have any brain disease; and (3) does not have any metabolic dysfunctions.

For the patient group, the inclusion criteria were: (1) subject was diagnosed with intractable medial temporal lobe epilepsy; (2) subject was not diagnosed with any other brain diseases or metabolic dysfunctions, and (3) subject underwent an SEEG exam.

Age, gender and other covariates were not treated as exclusion criteria. After exclusion criteria, there were 18 participants available from the healthy control group, and 24 epilepsy patients’ data. This was decided to be sufficient for our study as it was an approximately equal number in each group. As our study was exploratory of (previously unknown) aerobic glycolysis during the inter-ictal period, all data were used instead of selecting a reduced sample size for statistical purposes.

### Ethics

The study was conducted in accordance with the Helsinki Protocol and was approved by the Ethics Committee of ShanghaiTech University (IRB#2021-002) and the Ethics Committee of Shanghai Ruijin Hospital, Shanghai Jiao Tong University School of Medicine (No. 2016-123). For all included participants, written informed consent was provided.

### PET-MRI acquisition

Scanning was performed on a hybrid PET-MRI Siemens Biograph mMR scanner (Siemens Healthineers, Erlangen, Germany) using a 12-channel phase-array head coil. All subjects fasted for at least 6 hours before receiving an injection of 183.6 ± 32.0 MBq ^18^F-FDG while resting in a quiet, dimly lit room. They went through a simultaneous PET-MRI scan 40 min later, including static ^18^F-FDG-PET, sagittal T_1_-weighted imaging(T1WI), axial Pulsed Arterial Spin Labeling (PASL), axial transverse relaxation time (T_2_) mapping, and axial observed transverse relaxation time (T_2_*) mapping. PASL images were converted to quantitative cerebral blood-flow maps with the built-in QUantitative Imaging of Perfusion using a Single Subtraction (QUIPSS) method.^16^^,^^17^ For T1WI, we used Magnetization Prepared RApid Gradient Echo (MPRAGE) sequence, echo time (TE) = 2.44 ms, repetition time (TR) = 1900 ms, flip angle = 9º. For T_2_* mapping, we used Gradient Recalled Echo (GRE) sequence: TE = 2.46 / 4.92 / 7.38 / 9.84 ms, TR = 391 ms, flip angle = 25º. For T_2_ mapping, we used Spin Echo (SE) sequence: TE = 10.5 / 21.0 / 31.5 / 42.0 /52.5 / 63.0 ms, TR = 2000 ms, flip angle = 180º. For axial PASL: TE = 11 ms, TR = 2500 ms, flip angle = 90º.

### SEEG and surgery

Each epilepsy patient underwent SEEG electrode implantation navigated by their PET-MRI images to locate their possible epileptic focus. Guided by SEEG and PET-MRI results, clinicians diagnosed an epileptic focus location and resected this location immediately after the electrodes were removed. As this was the first study in epilepsy patients, epileptic foci were located using the existing methods including PET, anatomical MRI, and SEEG. Calibrated fMRI or OGI data were not used in this capacity. See Supplementary Methods 1 for detailed information.

### Data analysis

We retrospectively analysed PET-MRI imaging data after surgery had been completed and was evaluated. Data analysis was performed at ShanghaiTech University. As our study was exploratory of (previously unknown) aerobic glycolysis during the inter-ictal period, subjects’ images were not blinded between healthy controls and epilepsy patients, nor randomized left vs. right side.

#### Relative OGI calculation

**Figure 1:**
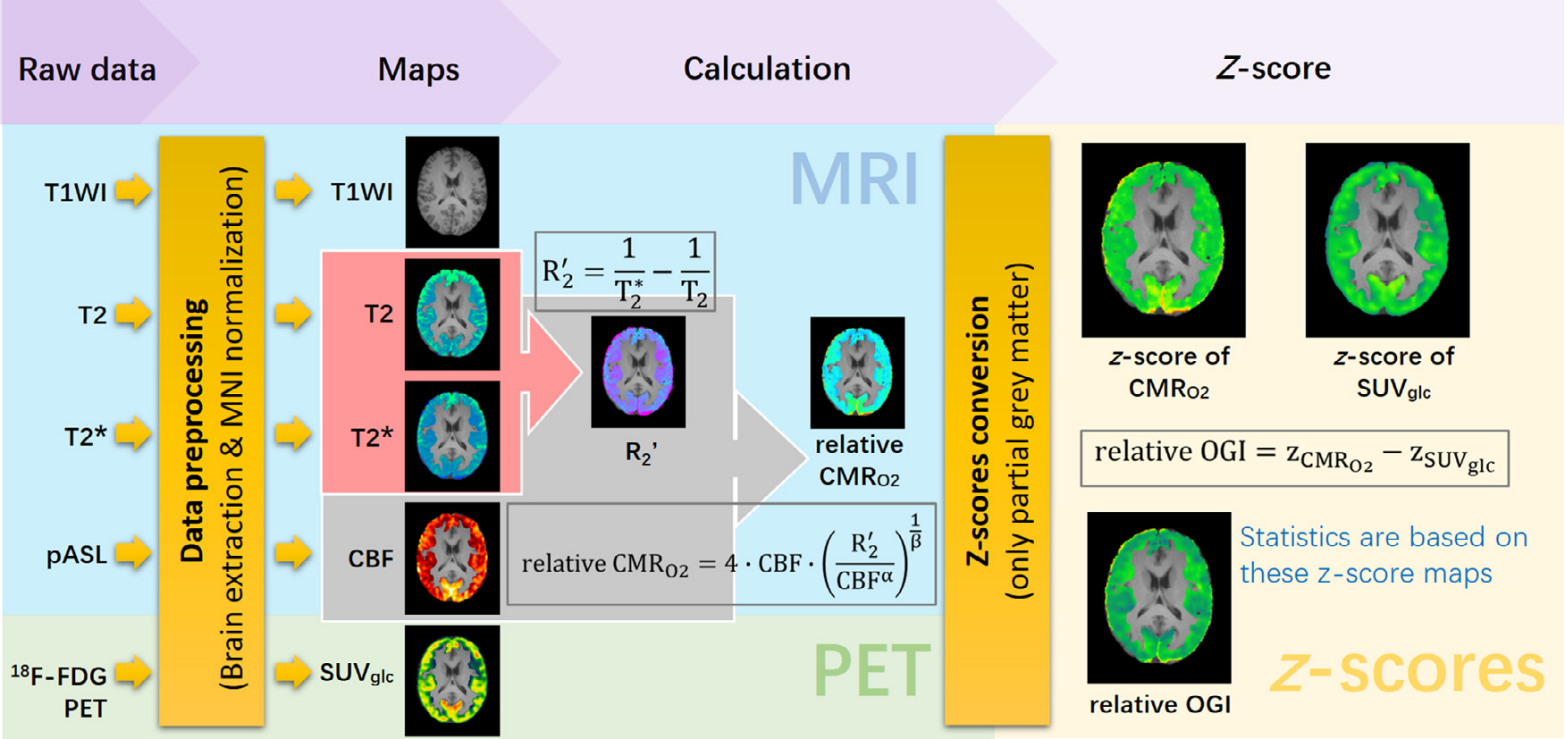
**Flowchart of relaxometry-based calibrated fMRI.** Different Modalities of PET-MRI data were used, including T1-weighted image (T1WI), T_2_, T_2_*, PASL and ^18^F-FDG PET. All images were registered into the MNI template before calculation and statistics. The final results of relative OGI are presented as *z*-scores.

The methods used to calculate relative OGI are summarized in Figure 1. After data preprocessing, we used MATLAB (2020b, The MathWorks, Inc., USA) to calculate the metabolic maps. All images were registered into a 1 mm MNI template and only selected regions of high grey matter volume (low partial volume of other tissues) were retained (Supplementary Table 1). Further calculations were done in MNI space.

We used a calibrated fMRI method that does not require gas exposure based on the previous work of Kida et al.^12^, Shu et al.^14^, and Xu et al.^11^ which we have used previously to calculate CMR_O2_ in mice.^11^ A summary of the theory is repeated in Supplementary Methods. The difference between relaxation rates R_2_* (or 1/T_2_*) and R_2_ (or 1/T_2_) is characterised by R_2_' (Eq. (1)), which is the part of the MRI signal that reflects non-homogeneous paramagnetic field alterations. This method uses R_2_' as a proxy for deoxyhemoglobin content. R_2_' is combined with cerebral blood flow (CBF) in a biophysical model^18^ to estimate the relative cerebral metabolic rate of oxygen (relative CMR_O2_) (Eq. (2), a full derivation is given in Supplementary Methods Equations S1-S6). In this work, we used the specific values for the blood oxygen-volume coupling parameter α and physical parameter β from Griffeth and Buxton (α = 0.14, β = 0.91).^19^

(1) $$R_{2}^{\prime }=R_{2}^{*}-R_{2}=\frac{1}{T_{2}^{*}}-\frac{1}{T_{2}}$$

(2) $$\text{relative}\;\text{CM}R_{O2}=4\cdot \text{CBF}\cdot {\left(\frac{R_{2}^{\prime }}{\text{CB}F^{\alpha }}\right)}^{\frac{1}{\beta }}$$

The standardized (glucose) uptake value (SUV_glc_) referenced to the body weight was calculated as described in Eq. (3):

(3) $$\text{SU}V_{\text{glc}}=\frac{\text{decay}\;\text{corrected}\;\text{actitvity}\;\text{concentration}}{\text{injected}\;\text{activity}\;/\;\text{body}\;\text{weight}}$$

The OGI is normally calculated as the ratio of oxygen metabolism to glucose metabolism and expressed on a scale between 0 (no glucose oxidized) and 6 (1 glucose + 6 O_2_ fully converted to 6 H_2_O + 6 CO_2_).^3^ However, since we are using relative measurements for both the CMR_O2_ (Eq. (2)), a normalizing method was used. Relative CMR_O2_ measurements were converted to *z*-scores by subtracting the mean of the grey matter and dividing the standard deviation, since the relative CMR_O2_ model was designed for grey matter.^18^ The *z*-score of each voxel can be calculated by Eq. (4):

(4) $$z=\frac{v-\mu }{\sigma }$$

where *z* is the z-score, *v* is the voxel value, *μ* is the mean value of all voxels, *σ* is the standard deviation.

When we treat all grey matter voxels in relative CMR_O2_ maps as a statistical population, this creates a hypothetical Gaussian distribution, *N*(0,1), and we apply the same conversion to SUV_glc_. If *z*-scores of relative CMR_O2_ and SUV_glc_ are subtracted, positive deviations indicate OGI that is greater than the whole-area grey matter, and negative deviations will indicate lower OGI/aerobic glycolysis than the whole-area grey matter:

(5) $$\text{relative}\;\text{OGI}=z_{\text{relative}\;\text{CM}R_{O2}}-z_{\text{SU}V_{glc}}$$

We can thus measure relative OGI in the epileptic foci to determine effects.

#### Statistics

The Shapiro-Wilk normality test was used to check the data distribution within each brain region and mean value distributions across brain regions. With normality confirmed, we used paired-sample, two-tailed *t*-tests to compare the differences between left and right hemispheres (healthy controls) or affected and contralateral hemispheres (patients) at the 0.05 confidence level. Two-sample, two-tailed *t*-tests were used to test the difference between two groups with different sizes. *p*-values are ***p ≤ 0.001, **p ≤ 0.01, *p ≤ 0.05. The Sequential Goodness of Fit (SGoF) method^20^ was used for multitest correction and all significance marked in the figures passed it.

### Role of Funders

This work was supported by ShanghaiTech University, the Shanghai Municipal Government, the National Natural Science Foundation of China Grant (No. 81950410637) and Shanghai Municipal Key Clinical Specialty (No. shslczdzk03403). F. H. and P. H. were supported by USA National Institute of Health grants (R01 NS-100106, R01 MH-067528). Z. W. was supported by the Key-Area Research and Development Program of Guangdong Province (2019B030335001), National Natural Science Foundation of China (No. 82151303), and National Key R&D Program of China (No. 2021ZD0204002). The funders had no active role in study design, data collection, data analyses, interpretation, or writing of this manuscript.

## Results

### Relative OGI is bilaterally symmetrical in healthy subjects

Per subject, the SUV_glc_ was determined from the [^18^F]FDG infusion using PET, whereas MRI provided independent measures of CBF and R_2_' to determine relative CMR_O2_. The SUV_glc_ and relative CMR_O2_ were then combined to determine relative OGI on a voxel-by-voxel basis. A summary of data processing method is shown in Figure 1.

**Figure 2:**
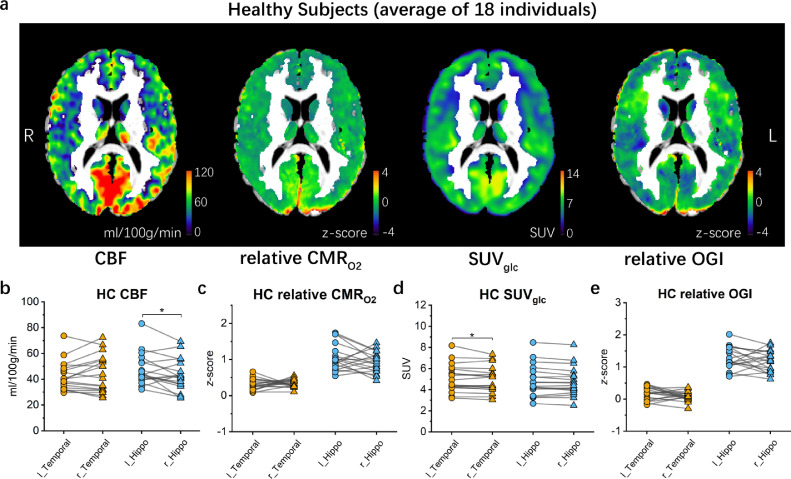
**Mean maps of healthy subjects and statistics on temporal lobe and hippocampus.** (**a**) Mean maps of CBF, relative CMR_O2_*z*-scores, SUV_glc_, and relative OGI *z*-scores of 18 healthy subjects. (**b-d**) Means of CBF (b), relative CMR_O2_*z*-score values (c), SUV_glc_ (d), and relative OGI (e) *z*-score values of left and right temporal lobe and hippocampus. Mean values of the left (circles) and right (triangles) brain regions within the same subject are connected by grey lines. The difference between population means in the left and right hippocampus in CBF is slightly significantly different from pair-sample *t*-tests, *p* = 0.039831. Abbreviations: l_temporal = left temporal lobe, r_temporal = right temporal lobe, l_hippo = left hippocampus, r_hippo = right hippocampus.

We found that the average *z*-score maps of the 18 healthy subjects were largely bilaterally symmetrical in the grey matter of the temporal lobe for all four modalities, CBF, relative CMR_O2_, SUV_glc_, relative OGI (Figure 2a) (and magnetic parameter R_2_' (Supplementary Figure 1)). The relative OGI in healthy subjects was similar to a previous study that measured absolute CMR_O2_ with ^15^O-O_2_ PET in healthy subjects (Supplementary Figure 2).^3^ There was also a small but significant difference in CBF in the hippocampus (pair-sample *t*-test, *df* = 17, *t* = 2.226, *p* = 0.039832, Figure 2b), and in SUV_glc_ in the temporal lobe (pair-sample *t*-test, *df* = 17, *t* = 2.122, *p* = 0.048817, Figure 2d). This asymmetry may be due to the comparatively older subjects who were available as controls.^21^ However, the temporal lobe and the hippocampus showed no significant differences in relative CMR_O2_ and relative OGI (pair-sample *t*-test, *df* = 17, *p* > 0.05) between the left and right hemispheres (Figure 2c and e). Results outside the temporal lobe and hippocampus are detailed in the supplementary information (Supplementary Figure 3).

### FDG-PET and relative OGI are asymmetrical in temporal lobe epilepsy patients

**Figure 3:**
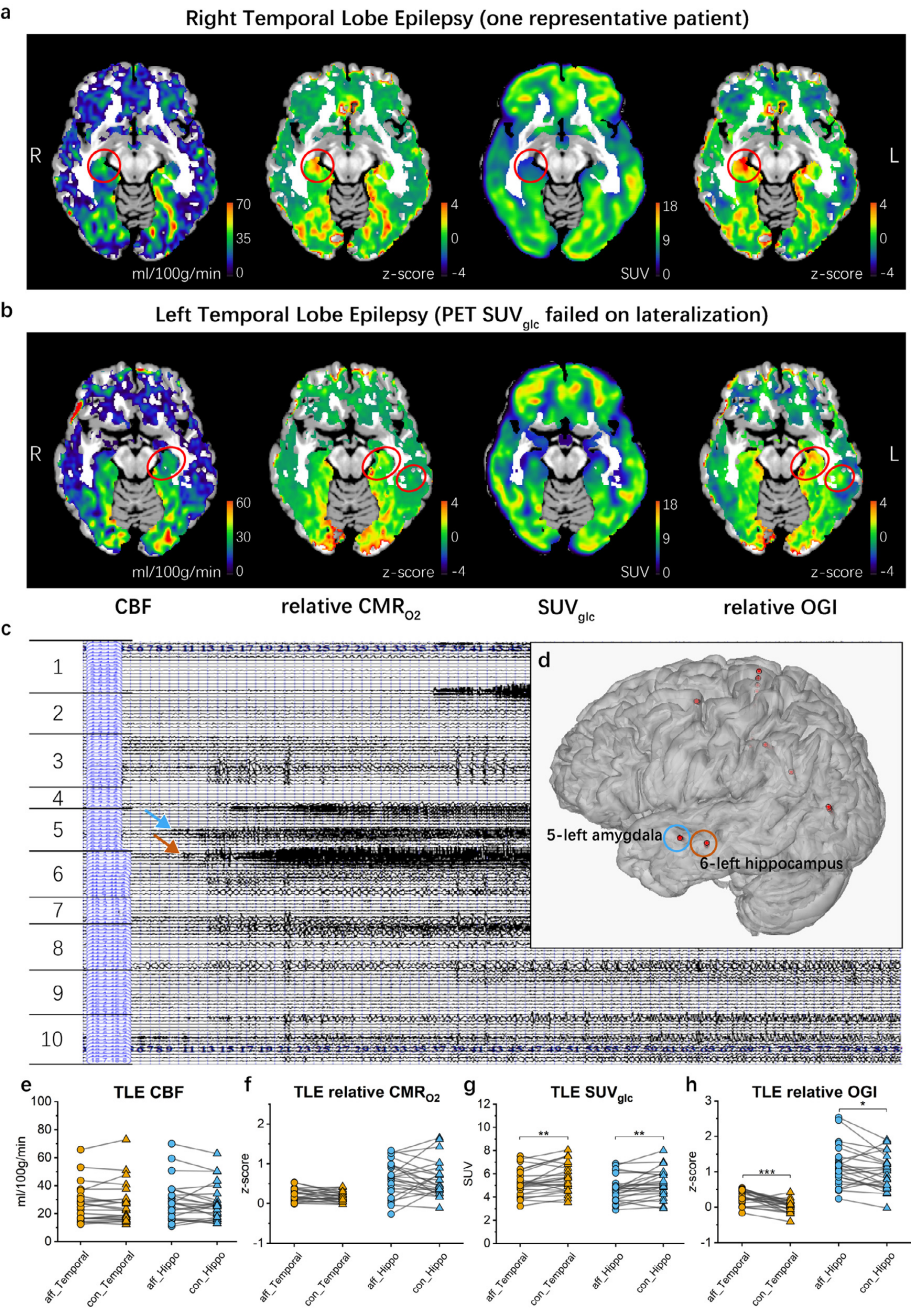
**Representative patients with temporal lobe epilepsy and statistics across different brain regions.** (**a**) Maps of CBF, relative CMR_O2_*z*-scores SUV_glc_ and relative OGI *z*-scores (as labelled in b) from one typical temporal lobe epilepsy patient where the SEEG, PET and OGI all identified the epileptic focus on the right side. The red circle indicates the abnormal values in the hippocampus on the affected right side of the brain. (**b**) Maps of CBF, relative CMR_O2_*z*-scores, SUV_glc_ and relative OGI *z*-scores from one temporal lobe epilepsy patient, in which PET results were inconclusive, but in which SEEG identified the epileptic focus on the left side. The red circle indicates the abnormal values from this study on the affected left side, which agree with the SEEG results. (**c**) SEEG trace of the seizure origin for the patient from **b**. Ictal SEEG recording showed that continuous, repetitive spikes arose from the left hippocampus (brown arrow) and left amygdala (blue arrow) and spread to the central area. The indices indicate the brain region as described in Supplementary Table 2. (**d**) Left lateral view of the reconstructed 3D MRI brain image of the patient from **b**. The positions of the right hemisphere electrodes are shown in red, co-registered with the MRI image. The red dots circled in blue (left amygdala) and brown (left hippocampus) show the position of the electrodes that detected abnormal discharges. (**e-h**) Mean CBF (**e**), relative CMR_O2_*z*-score values (f), SUV_glc_ (**g**)*,* and relative OGI (h) *z*-score values of the left and right temporal lobe and hippocampus. The differences of the population mean on the left and right brain regions in SUV_glc_ and relative OGI are significantly different using pair-sample *t*-tests, **0.001<*p*<0.01, ***p<0.001. In SUV_glc_ (**g**), *p* = 0.0098595 for temporal lobe, *p* = 0.0091322 for hippocampus. In relative OGI (**f**), *p* = 1.5594×10^−5^ for temporal lobe, *p* = 0.029193 for hippocampus.

The data processing methods described above were also applied to patients’ data. For temporal lobe epilepsy patients, there are significant differences between the ipsilateral (affected) and contralateral (unaffected) hemispheres, as shown in the representative right and left temporal lobe epilepsy subjects in Figure 3a and b, respectively. For the subject shown in Figure 3a, SUV_glc_ located the correct side as verified by SEEG, and the OGI results agreed, being higher in the right temporal lobe of the affected side than the unaffected side. However, for the subject shown in Figure 3b, the results from SUV_glc_ failed to locate the side of the epileptic focus, yet relative OGI was higher on the left side than the right side, as verified by the SEEG traces from this patient (Figure 3c and d). Results outside the temporal lobe, and magnetic relaxation parameter R_2_’ are detailed in the supplementary information (Supplementary Figure 1 and 4). Among 24 patients, there was no significant difference between affected and contralateral sides in CBF (Figure 3e) and relative CMR_O2_ (Figure 3f). However, SUV_glc_ showed significantly lower values in the temporal lobe (pair-sample *t*-test, *df* = 23, *t* = -2.813, *p* = 0.0098595) and hippocampus (pair-sample *t*-test, *df* = 23, *t* = -2.847, *p* = 0.0091322) on the affected side than the unaffected side (Figure 3g), and relative OGI showed significantly higher values in the temporal lobe (pair-sample *t*-test, *df* = 23, *t* = 5.444, *p* = 1.5594×10^−5^) and hippocampus (pair-sample *t*-test, *df* = 23, *t* = 2.326, *p* = 0.029193) on the affected side versus the unaffected side (Figure 3h).

### Relative OGI may offer complementary information to FDG-PET

**Figure 4:**
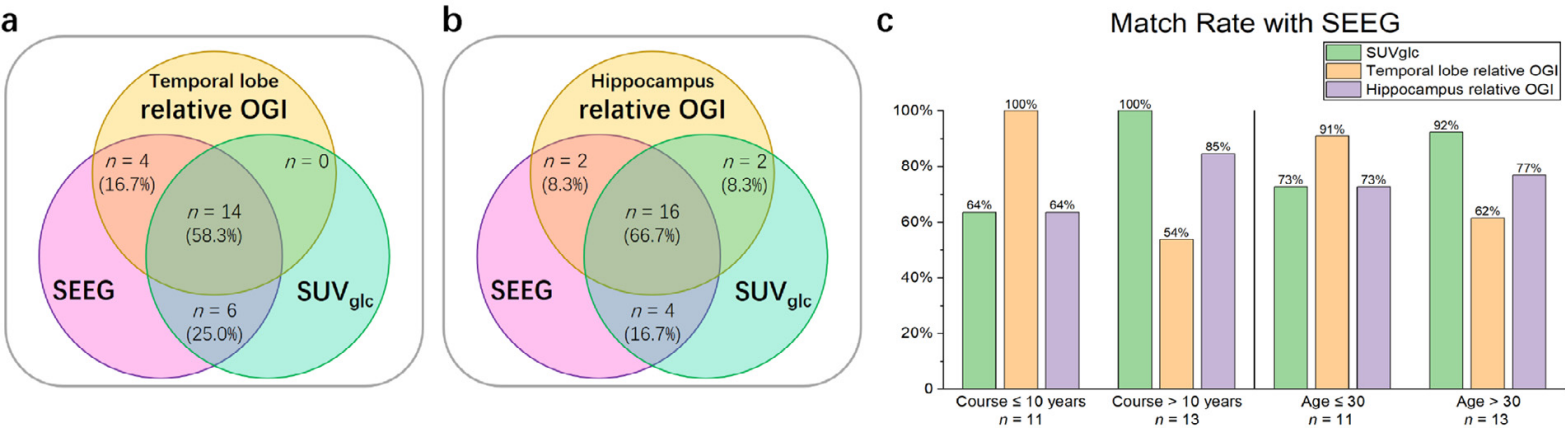
**Match rate among modalities.** Venn diagram of match rate for relative OGI, SUV_glc_, and SEEG, based on (**a**) temporal lobe and (**b**) hippocampus. The number *n* of diagnoses in each group (and the total percentage of patients in this study) is shown in respective areas. (**c**) Comparisons of match rate between SUV_glc_ and SEEG or relative OGI and SEEG in a relatively shorter course under 10 years of epilepsy, or younger age under 30 years old, and longer course over 10 years, or older age over 30 years old, respectively.

To test the potential of relative OGI as a complementary method for epilepsy lateralization, we calculated the match rate of predicted brain hemisphere for the epileptic focus between SUV_glc_, relative OGI, and SEEG (Figure 4a and b). For the temporal lobe, while 83.3% of SUV_glc_ tests and 75.0% relative OGI tests matched SEEG when only the single metric was considered, at least one out of the two metrics, SUVglc or relative OGI, always matched SEEG for all 24 patients. For the hippocampus, the result is similar, but 8.3% of tests in which relative OGI and SUVglc matched each other but not SEEG. While preliminary, our results suggest that relative OGI offers diagnostic information beyond what oxygen or glucose imaging alone offers, in particular for cases where glucose imaging through PET may be inconclusive.

Interestingly, in a posthoc analysis, we found that while PET had a better match rate with SEEG overall, relative OGI has a better match rate with SEEG in the temporal lobe in both patients in the earlier course of the disease (had epilepsy for fewer years) and patients of younger age. (Note that, there is substantial overlap between these groups, as younger patients have had the disease for less time.) Alternately, in the hippocampus, OGI may match SEEG better in the latter groups for both course and age (Figure 4c). However, more data are needed for definitive conclusions, particularly from patients where PET and SEEG do not match.

## Discussion

To improve epilepsy diagnosis and to understand how it differs across patients, new imaging methods that can act as biomarkers are needed.^1^ We used a simultaneous PET-MRI scanner to measure both oxygen metabolism with calibrated fMRI and glucose metabolism with FDG-PET. We found that calculating OGI from these data revealed more information than either oxygen or glucose metabolism alone, because the glycolytic shunt is indicative of biosynthesis pathways.^22^ Thus, our detection of increased OGI during the inter-ictal phase on the ipsilateral hemisphere to epileptic foci may help clinicians understand individual patients’ aetiology. While metabolic networks based on FDG-PET have sometimes been considered an objective biomarker for the assessment of certain treatments,^23^ OGI appears to provide complementary information to glucose uptake, which may help locate or rank epileptic foci. In addition, of the three patients with poor outcomes (Engel class II or III), two of them had a bilateral disruption in relative OGI, whereas SEEG and SUV_glc_ in those patients were only disrupted on one side (Supplemental Table S3).

### Hypothetical source of increased OGI during inter-ictal periods

Metabolic disturbances at the epileptic focus are known to persist for 90 minutes to 2 hours after seizures^8^ ^24^ and return to normal within 24 hours^25^. In our study, there was a delay of at least 24 hours between the most recent seizure and imaging. Thus, our observations are firmly within the inter-ictal period. While the present study is limited by not having direct measurements of metabolites, we can hypothesize as to the source of increased OGI we observed.

At present, measuring glucose use in the brain with ^18^F-FDG is a prominent method of locating epileptic foci and classifying epilepsy types^26^ during the inter-ictal period. This is because the epileptic focus, versus surrounding tissue and the contralateral equivalent region, has greater glucose metabolism during seizures but lower during the inter-ictal period^1^^,^^9^. While OGI is likely lower during seizures than the inter-ictal period due to the increase in lactate efflux,^8^ we observed higher OGI during the inter-ictal period on the side of the epileptic focus relative to the other side. Relative CMR_O2_ was not significantly different. This supports an aetiology of the inter-ictal period being marked by decreased glucose metabolism yet having the same oxygen supply. However, as OGI seems to provide additional information to glucose measurements alone (e.g., Figure 4) non-significant oxygen metabolism changes may exist in concert with glucose changes to alter local aerobic glycolysis.

An important question remains: why would the relative level of glycolysis be lower in the epileptic focus than in healthy tissue in the inter-ictal period? In healthy subjects, Fox, et al. previously observed that OGI will also decrease in healthy brain tissue when the immediate energy need is high and the slower oxidative phosphorylation cannot keep pace with glycolysis.^7^ Under normal conditions this would result in lactate efflux to balance the carbon load^27^^,^^28^ or increase the ratio of NADH to NAD+.^29^, ^30^, ^31^ However, under epilepsy, the outpacing of oxidative phosphorylation by glycolysis would be even more excessive.^8^

Metabolic stress on the neural tissue at the epileptic focus triggers cellular and sub-cellular changes which continue into the inter-ictal period. As these changes include altered shapes of mitochondria, affecting oxygen metabolism, and more astrocytes, affecting pyruvate transport^32^ it is possible that these alterations could allow increased oxygen metabolism during the inter-ictal period. Interestingly, some subjects did have higher relative CMR_O2_ on the affected hemisphere than the other hemisphere, though this was not significant on a group level, and the interhemispheric difference of OGI exceeded that of relative CMR_O2_ (Figure 3). If higher relative CMR_O2_ at the epileptic focus (relative to contralateral or surrounding tissue) does exist in some patients, this would support this hypothesis; however, further study is needed.

### OGI and the course of epilepsy

The comparison between averages from subjects who had suffered from epilepsy for under ten years, versus those who had suffered for over ten years, suggests that, on average, over the course of epilepsy there is a change from OGI at seizure foci being more lateralized (subjects with epilepsy under ten years) to glucose hypometabolism being more lateralized (subjects with epilepsy over ten years). While any mechanism would be speculation at this point, this suggests that OGI may be a better biomarker for early epilepsy while its effectiveness decreases over time. However, more data, particularly longitudinal, are needed to distinguish the metabolic changes caused by epilepsy or ageing.^33^ Such longitudinal effects may not be possible to study ethically in human subjects as it would require a longitudinal study on untreated epilepsy patients, thus animal models may prove helpful here.

### Outlook

The source of the altered metabolism during the inter-ictal period also requires further study, e.g., whether glucogenesis, e.g., during the ictal period, could be contributing to brain glucose metabolism and also altering neurotransmitter balance, such as glutamate vs. *gamma*-Aminobutyric acid (GABA) and glutamate vs. glutamine. Magnetic resonance spectroscopy of endogenous hydrogen (1H nucleus) has been used in epilepsy patients previously^25^ and is feasible for investigating glutamate vs. GABA^34^, and similar techniques on exogenous carbon (^13^C) for glutamate vs. glutamine cycling^35^. Our study provides further support for the importance of glycolysis vs. oxidative metabolism differences in epilepsy, in particular as it suggests the seizure foci may commit less glycolysis during the inter-ictal period than surrounding brain tissue. As several therapies for reducing relative glycolysis levels have been used for epilepsy, e.g. ketogenic diet^36^ or 2-deoxy-D-glucose administration^37^, it would be interesting to use OGI to monitor such treatments for effectiveness.

## Conclusion

Our OGI method based on aerobic glycolysis is fast, simple and feasible. It could conceivably be implemented in any hospital with a combined PET-MRI system. As our method allows OGI imaging within a single session, it also has potential for use in other diseases where aerobic glycolysis may be disrupted; in particular, effects of previous malnutrition^5^, age-related dementia,^38^ Huntington's Disease,^6^ and metabolic disorders. While further work is necessary, our method provides OGI as a new biomarker for epilepsy, and provides insight into the metabolic effects of epilepsy in inter-ictal periods.

## Contributors

G.J.T. conceived of the main concept and guided the project; Q.Q. and M.Z. contributed to the study design; M.Z., H.M., X.H., and X.L. performed PET-MRI scanning; W.L. performed SEEG and data analysis; Q.Q., S.Z., and M.X. processed and analysed the imaging data; Q.Q. and S.Z. have directly accessed and verified the underlying data, and conducted the statistics; M.Z. collated pathological information; M.L. helped with MR sequences and technique support; P.H., F.H., B.L., Z.W., and R.C.S. helped with critical advice and discussion. G.J.T., Q.Q., and M.Z. drafted the manuscript and all authors discussed the results and contributed to the writing of the paper. Q.Q. and M.Z. contributed equally to this work as co-first authors. G.J.T. and B.L. are co-corresponding authors and are responsible for the decision to submit the manuscript. All authors read and approved the final version of the manuscript.

## Data sharing

The datasets generated during and/or analysed during the current study are available from OpenNeuro (doi:https://doi.org/10.18112/openneuro.ds004054.v1.0.0). All in-house developed MATLAB scripts used for data processing and statistics are available on reasonable request.

## Declaration of Interests

M.L. is an employee of Siemens Healthineers Ltd., Shanghai, China. G.J.T. was supported by the Chinese Neuroscience Society to give a presentation at its annual meeting, Sept. 2021. Other authors have no conflicts of interest to disclose as described by eBioMedicine.

## Appendix Supplementary materials

Image, application 1

Image, application 2

## References

[1] Kini L.G., Gee J.C., Litt B. (2016). Computational analysis in epilepsy neuroimaging: a survey of features and methods. NeuroImage Clin.

[2] Guedj E., Bonini F., Gavaret M. (2015). 18FDG-PET in different subtypes of temporal lobe epilepsy: SEEG validation and predictive value. Epilepsia.

[3] Hyder F., Herman P., Bailey C.J. (2016). Uniform distributions of glucose oxidation and oxygen extraction in gray matter of normal human brain: no evidence of regional differences of aerobic glycolysis. J Cereb Blood Flow Metab.

[4] Vaishnavi S.N., Vlassenko A.G., Rundle M.M., Snyder A.Z., Mintun M.A., Raichle M.E. (2010). Regional aerobic glycolysis in the human brain. Proc Natl Acad Sci U S A.

[5] Mehta S., Kalsi H.K., Nain C.K., Menkes J.H. (1977). Energy Metabolism of brain in human protein-calorie malnutrition. Pediatr Res.

[6] Powers W.J., Videen T.O., Markham J. (2007). Selective defect of *in vivo* glycolysis in early Huntington's disease striatum. Proc Natl Acad Sci.

[7] Fox P.T., Raichle M.E., Mintun M.A., Dence C. (1988). Nonoxidative glucose consumption during focal physiologic neural activity. Science.

[8] During M.J., Fried I., Leone P., Katz A., Spencer D.D. (1994). Direct measurement of extracellular lactate in the human hippocampus during spontaneous seizures. J Neurochem.

[9] Hajek M., Antonini A., Leenders K.L., Wieser H.G. (1993). Mesiobasal versus lateral temporal lobe epilepsy: metabolic differences in the temporal lobe shown by interictal 18F-FDG positron emission tomography. Neurology.

[10] Shu C.Y., Sanganahalli B.G., Coman D. (2016). Chapter 5 - new horizons in neurometabolic and neurovascular coupling from calibrated fMRI. Progress in Brain Research.

[11] Xu M., Bo B., Pei M. (2021). High-resolution relaxometry-based calibrated fMRI in murine brain: metabolic differences between awake and anesthetized states. J Cereb Blood Flow Metab.

[12] Kida I., Kennan R.P., Rothman D.L., Behar K.L., Hyder F. (2000). High-resolution CMR(O2) mapping in rat cortex: a multiparametric approach to calibration of BOLD image contrast at 7 Tesla. J Cereb Blood Flow Metab.

[13] Hyder F., Kida I., Behar K.L., Kennan R.P., Maciejewski P.K., Rothman D.L. (2001). Quantitative functional imaging of the brain: towards mapping neuronal activity by BOLD fMRI. NMR Biomed.

[14] Shu C.Y., Herman P., Coman D. (2016). Brain region and activity-dependent properties of M for calibrated fMRI. Neuroimage.

[15] Engel J.J. (1993). Surgical Treatment of the Epilepsies.

[16] Alsop D.C., Detre J.A., Golay X. (2015). Recommended implementation of arterial spin-labeled perfusion MRI for clinical applications: a consensus of the ISMRM perfusion study group and the European consortium for ASL in dementia. Magn Reson Med.

[17] Wong E.C., Buxton R.B., Frank L.R. (1998). Quantitative imaging of perfusion using a single subtraction (QUIPSS and QUIPSS II). Magn Reson Med.

[18] Hoge R.D., Atkinson J., Gill B., Crelier G.R., Marrett S., Pike G.B. (1999). Investigation of BOLD signal dependence on cerebral blood flow and oxygen consumption: the deoxyhemoglobin dilution model. Magn Reson Med.

[19] Griffeth V.E.M., Buxton R.B. (2011). A theoretical framework for estimating cerebral oxygen metabolism changes using the calibrated-BOLD method: modeling the effects of blood volume distribution, hematocrit, oxygen extraction fraction, and tissue signal properties on the BOLD signal. Neuroimage.

[20] Carvajal-Rodriguez A., de Una-Alvarez J., Rolan-Alvarez E. (2009). A new multitest correction (SGoF) that increases its statistical power when increasing the number of tests. BMC Bioinf.

[21] He X., Qin W., Liu Y. (2013). Age-related decrease in functional connectivity of the right fronto-insular cortex with the central executive and default-mode networks in adults from young to middle age. Neurosci Lett.

[22] Dienel G.A. (2019). Brain glucose metabolism: integration of energetics with function. Physiol Rev.

[23] Ge J., Wang M., Lin W. (2020). Metabolic network as an objective biomarker in monitoring deep brain stimulation for Parkinson's disease: a longitudinal study. EJNMMI Res.

[24] Petroff O.A.C., Prichard J.W., Ogino T., Avison M., Alger J.R., Shulman R.G. (1986). Combined 1H and 31P nuclear magnetic resonance spectroscopic studies of bicuculline-induced seizures in vivo. Ann Neurol.

[25] Breiter S.N., Arroyo S., Mathews V.P., Lesser R.P., Bryan R.N., Barker P.B. (1994). Proton MR spectroscopy in patients with seizure disorders. Am J Neuroradiol.

[26] Li Y., Feng J., Zhang T. (2021). Brain metabolic characteristics distinguishing typical and atypical benign epilepsy with centro-temporal spikes. Eur Radiol.

[27] Dienel G.A., McKenna M.C. (2014). A dogma-breaking concept: glutamate oxidation in astrocytes is the source of lactate during aerobic glycolysis in resting subjects. J Neurochem.

[28] Sonnewald U. (2014). Glutamate synthesis has to be matched by its degradation – where do all the carbons go?. J Neurochem.

[29] Hertz L., Chen Y. (2017). Integration between glycolysis and glutamate-glutamine cycle flux may explain preferential glycolytic increase during brain activation, requiring glutamate. Front Integr Neurosci.

[30] Hertz L., Rothman D.L., Schousboe A, Sonnewald U (2016). The Glutamate/GABA-Glutamine Cycle: Amino Acid Neurotransmitter Homeostasis.

[31] Hyder F., Rothman D.L., Bennett M.R. (2013). Cortical energy demands of signaling and nonsignaling components in brain are conserved across mammalian species and activity levels. Proc Natl Acad Sci.

[32] Ingvar M., Söderfeldt B., Folbergrová J., Kalimo H., Olsson Y., Siesjö B.K. (1984). Metabolic, circulatory, and structural alterations in the rat brain induced by sustained pentylenetetrazole seizures. Epilepsia.

[33] Zhang H., Wu P., Ziegler S.I. (2017). Data-driven identification of intensity normalization region based on longitudinal coherency of 18F-FDG metabolism in the healthy brain. Neuroimage.

[34] Igarashi H., Ueki S., Ohno K., Ohkubo M., Suzuki Y. (2017). Magnetic resonance imaging of neurotransmitter-related molecules. J Nippon Med Sch.

[35] Rothman D.L., De Feyter H.M., de Graaf R.A., Mason G.F., Behar K.L. (2011). 13C MRS studies of neuroenergetics and neurotransmitter cycling in humans. NMR Biomed.

[36] Vidali S., Aminzadeh S., Lambert B. (2015). Mitochondria: the ketogenic diet—a metabolism-based therapy. Int J Biochem Cell Biol.

[37] Stafstrom C.E., Roopra A., Sutula T.P. (2008). Seizure suppression via glycolysis inhibition with 2-deoxy-D-glucose (2DG). Epilepsia.

[38] Lajoie I., Nugent S., Debacker C. (2017). Application of calibrated fMRI in Alzheimer's disease. NeuroImage Clin.

