# Supplementary material for: Aerobic glycolysis imaging of epileptic foci during the inter-ictal period
Source: eBioMedicine. 2022 Apr 15;79:104004. doi: 10.1016/j.ebiom.2022.104004 (PMC9035653; doi:10.1016/j.ebiom.2022.104004)
Supplement: Supplementary file 1 [file mmc1.docx]

**Caption for Supplementary Material**

**1. Supplementary Information for Aerobic glycolysis imaging of epileptic foci during the inter-ictal period**

file name: Supplementary_R1_Clean.pdf

including:

Supplementary Table 1

Supplementary Table 2

Supplementary Table 3

Supplementary Figure 1

Supplementary Figure 2

Supplementary Figure 3

Supplementary Figure 4

Supplementary Methods

References
