# Supplementary material for: Aerobic glycolysis imaging of epileptic foci during the inter-ictal period
Source: eBioMedicine. 2022 Apr 15;79:104004. doi: 10.1016/j.ebiom.2022.104004 (PMC9035653; doi:10.1016/j.ebiom.2022.104004)
Supplement: Supplementary file 2 [file mmc2.pdf]

Supplementary Information for

Aerobic glycolysis imaging of epileptic foci during the inter-ictal period

Miao Zhang[1,†], Qikai Qin (秦琪凯)[2,3,4,†], Shuning Zhang[2,3,4], Wei Liu[5], Hongping Meng[1], Mengyang Xu[2,3,4,6], Xinyun Huang[1], Xiaozhu Lin[1], Mu Lin[7], Peter Herman[8,9,10], Fahmeed Hyder[8,9,10,11], Raymond C. Stevens[2,3], Zheng Wang[12], Biao Li[1,13,*], and Garth J. Thompson[2,*].

[†]**These authors contributed equally to this work.**

Correspondence to: Biao Li and Garth J. Thompson

Biao Li

Department of Nuclear Medicine, Ruijin Hospital, Shanghai Jiao Tong University School of Medicine, No.197 Rui Jin 2nd Road, Shanghai, 200025, China. lb10363@rjh.com.cn

Garth J. Thompson

iHuman Institute, ShanghaiTech University, 393 Middle Huaxia Road, Pudong, Shanghai, 201210, China. gthompson@shanghaitech.edu.cn

Supplementary contents

No.	Title	Page
Supplementary Table 1	Brain region parcellation based on Yale brain template	2
Supplementary Table 2	SEEG indexes of electrodes implantation	4
Supplementary Table 3	Patients' diagnosis and postsurgical outcomes	5
Supplementary Figure 1	R2' maps	6
Supplementary Figure 2	Relative OGI across brain regions of healthy subjects	7
Supplementary Figure 3	Statistics across brain regions of healthy subjects	8
Supplementary Figure 4	Statistics across brain regions of temporal lobe epilepsy patients	9
Supplementary Methods		10
References		12

Supplementary Table 1. Brain region parcellation based on Yale brain template

Index in Yale template	Description
**Parietal lobe (1/101)**	
1/101	Primary Somatosensory Cortex
5/105	Somatosensory Association Cortex
7/107	Somatosensory Association Cortex, Praecuneus
39/139	Angular Gyrus, (part of Wernicke area)
40/140	Supramarginal Gyrus, (part of Wernicke area)
**Temporal lobe (2/102)**	
20/120	Inferior Temporal Gyrus
21/121	Middle Temporal Gyrus
22/122	Superior Temporal Gyrus, (caudal part is the Wernicke area)
37/137	Fusiform Gyrus
38/138	Temporopolar Area
41/141	Primary and Auditory Association Cortex
**Frontal lobe (3/103)**	
4/104	Primary Motor Cortex
6/106	Premotor Cortex, Supplementary Motor Cortex
8/108	includes Frontal Eye Field
9/109	Dorsolateral Prefrontal Cortex
10/110	Anterior Prefrontal Cortex
11/111	Orbitofrontal Area
25/125	Subgenual Cortex
44/144	Pars Opercularis, part of Broca area
45/145	Pars Triangularis Broca area
46/146	Dorsolateral Prefrontal Cortex
47/147	Inferior Prefrontal Gyrus
**Insular cortex (4/104)**	
13/113	Insular Cortex
**Occipital lobe (5/105)**	
17/117	Primary Visual Cortex (V1)
18/118	Secondary Visual Cortex (V2)
19/119	Associative Visual Cortex (V3)
**Hippocampus (8/108)**	
54/154	Hippocampus
**Other brain regions**	
23/123	Ventral Posterior Cingulate Cortex
24/124	Ventral Anterior Cingulate Cortex
30/130	Part of Cingulate Cortex
31/131	Dorsal Posterior Cingulate Cortex

Continued Supplementary Table 1.

32/132	Dorsal Anterior Cingulate Cortex
34/134	Anterior Entorhinal Cortex (on the parahippocampal gyrus)
36/136	Parahippocampal Cortex (on the parahippocampal gyrus)
48/148	Caudate
49/149	Putamen
50/150	Thalamus
51/151	Globus Pallidus
52/152	Nucleus Accumbens
53/153	Amygdala
55/155	Hypothalamus

The parcellation we used (in bold font) is based on a finer Yale template.[1] Description of brain regions is in terms of neuroanatomical identities, which are similar to Brodmann areas. The index of these brain regions in mask file for this study are described in parentheses. The double-digit numbers indicate the left hemisphere, while the three-digit numbers indicate the right hemisphere. These regions were used as they had high grey matter volume and low partial volume of other tissues.

Supplementary Table 2. SEEG indexes of electrodes implantation

Index	Brain region
1	Right Amygdala-Temporal lobe
2	Right Hippocampus-Temporal lobe
3	Left Occipital lobe
4	Left Posterior cingulate gyrus-Supplementary motor area
5	Left Amygdala-Temporal lobe
6	Left Hippocampus-Temporal lobe
7	Left Frontal lobe(F1-F2)
8	Left Posterior cingulate gyrus-Angular gyrus
9	Right postcentral gyrus
10	Left Posterior cingulate gyrus-Supramarginal gyrus

There were 10 SEEG electrodes implanted into the patient of Figure 3c and 3d as shown in the main text. The above table lists the locations of these 10 electrodes.

Supplementary Table 3. Patients' diagnosis and postsurgical outcomes

Subject	Postsurgical outcome	Age	Course of epilepsy	SEEG	SUV_{glc}	relative OGI
TLE08	Engel Class Ⅲ	34	20 years	Left	Left	Bilateral
TLE17	Engel Class Ⅱ	23	19 years	Left	Left	Bilateral
TLE19	Engel Class Ⅲ	13	10 years	Left	Bilateral	Left

There are three patients with poor outcome (Engel class II or III). Two of them had a bilateral disruption in relative OGI of temporal lobe, whereas SEEG and SUV_{glc} in those patients were only disrupted on one side.

Supplementary Figure 1. R_2' maps

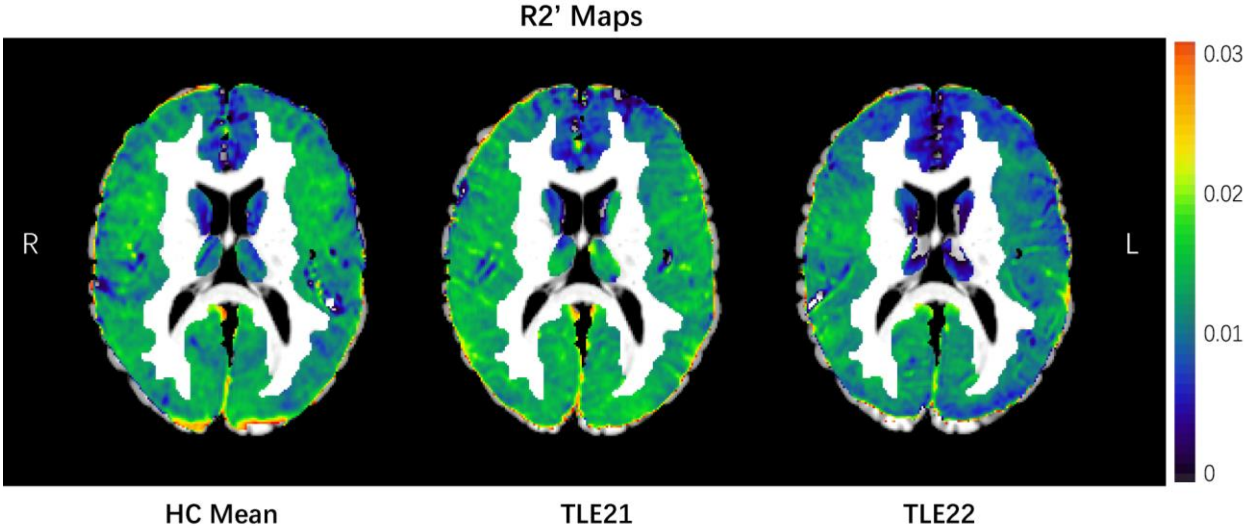

Supplementary Figure 1. R_2' maps. Averaged R_2' map of 18 healthy subjects (HC Mean), R_2' map from the right temporal lobe of the epilepsy patient in Figure 3a (TLE21), and R_2' map from the left temporal lobe of the epilepsy patient in Figure 3b (TLE22) are from left to right, respectively.

Supplementary Figure 2. Relative OGI across brain regions of healthy subjects

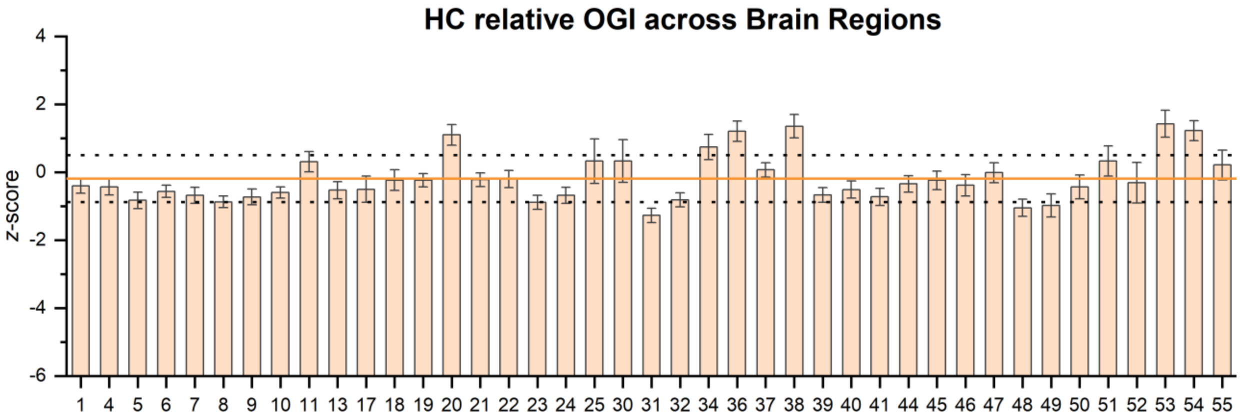

Supplementary Figure 2. Relative OGI across brain regions of healthy subjects. Mean relative OGI values across 41 brain regions (Supplementary Table 1) in healthy controls of our PET/MR study are shown. The black dashed horizontal lines are ±SD around the mean of all voxels, within which most brain region mean values fall in. In an overall view, relative OGI are similar to Hyder et al., 2016.[1]

Supplementary Figure 3. Statistics across brain regions of healthy subjects

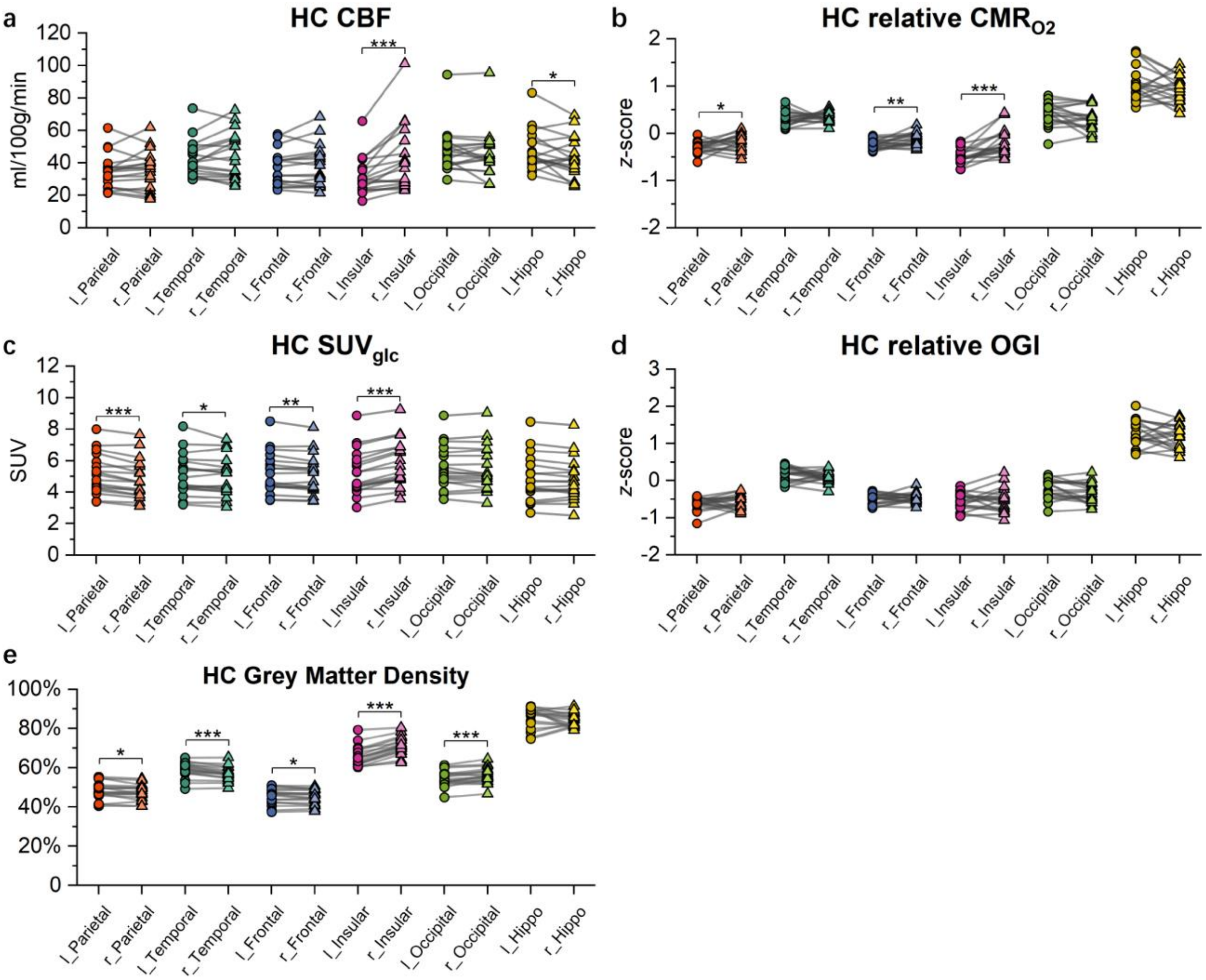

Supplementary Figure 3. Statistics across brain regions of healthy subjects. Statistics on (a) CBF, (b) relative CMR_{O2} *z*-scores, (c) SUV_{glc} and (d) relative OGI *z*-scores in healthy subjects. Mean values of the left (circle) and right (triangle) brain regions within the same subject are connected by grey lines. For CBF, relative CMR_{O2} *z*-scores and SUV_{glc}, even in healthy subjects, there are significant differences between left and right side in certain brain regions from pair-sample, two-tailed *t*-tests, $n = 18$, $*p < 0.05$, $**p < 0.01$, $***p < 0.001$. In (a) CBF, $p = 9.5414\times10^{-4}$ for insular cortex, $p = 0.039831$ for hippocampus. In relative (b) CMR_{O2}, $p = 0.042607$ for parietal lobe, $p = 0.0029475$ for frontal lobe, $p = 1.3299\times10^{-4}$ for insular cortex. In (c) SUV_{glc}, $p = 1.7769\times10^{-5}$ for parietal lobe, $p = 0.048817$ for temporal lobe, $p = 0.0013620$ for frontal lobe, $p = 2.6901\times10^{-9}$ for insular cortex. However, this was not observed in relative OGI, suggesting relative OGI as a potential stable biomarker. (e) Grey matter density of different brain region. *P*-values are 0.039466, 9.2402×10^{-5}, 0.048786, 1.29938×10^{-8}, 6.1007×10^{-4}, and 0.20478, respectively. For all tests above, sequential Goodness of Fit (SGoF) was used to test for Type I errors. Statistical families were the measurement modalities. All $p < 0.05$ remained significant after SGoF correction. In the x-axis label, “l” and “r” stand for left and right, respectively. “Parietal” Parietal lobe, “Temporal” Temporal lobe, “Frontal” Frontal lobe, “Insular” Insular cortex, “Occipital” Occipital lobe, “Hippo” hippocampus.

Supplementary Figure 4. Statistics across brain regions of temporal lobe epilepsy patients

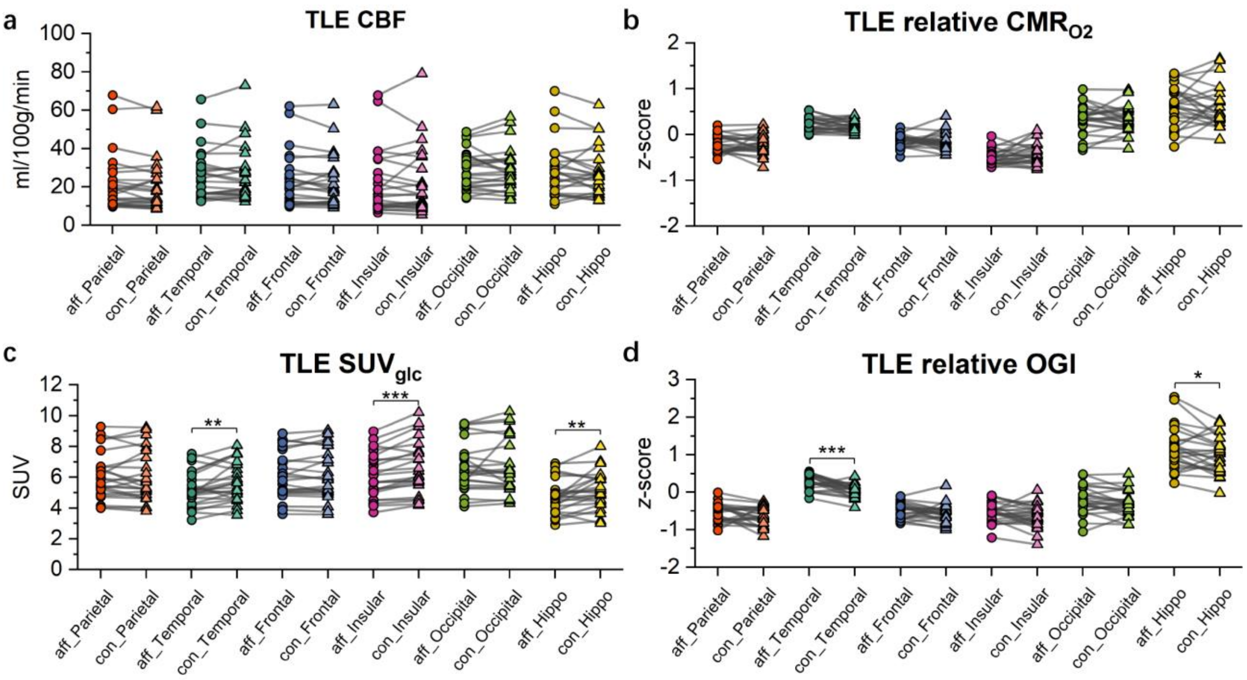

Supplementary 4. Statistics across brain regions of temporal lobe epilepsy patients. Statistics on (a) CBF, (b) relative CMR_{O2} *z*-scores (c) SUV_{glc} and (d) relative OGI *z*-scores in temporal lobe epilepsy patients. Mean values of the left (circle) and right (triangle) brain regions within the same subject are connected by grey lines. For SUV_{glc} and relative OGI, there are significant difference between left and right hemispheres in some brain regions from pair-sample, two-tailed *t*-tests, $n = 24$, *$p<0.05$, **$p<0.01$, ***$p<0.001$. In (c) SUV_{glc}, $p = 0.0098595$ for temporal lobe, $p = 4.4271\times10^{-5}$ for insular cortex, $p = 0.0091322$ for hippocampus. In (d) relative OGI, $p = 1.5594\times10^{-5}$ for temporal lobe, $p = 0.029193$ for hippocampus. SUV_{glc} and relative OGI results are similar to each other. For all tests above, sequential Goodness of Fit (SGoF) was used to test for Type I errors. Statistical families were the measurement modalities. All $p < 0.05$ remained significant after SGoF correction. In the x-axis label, "aff" and "con" stand for affected and contralateral, respectively. "Parietal" Parietal lobe, "Temporal" Temporal lobe, "Frontal" Frontal lobe, "Insular" Insular cortex, "Occipital" Occipital lobe, "Hippo" hippocampus.

Supplementary Methods

1. Surgery

1.1. SEEG electrode implantation navigated by hybrid PET/MRI imaging

Following pre-surgical evaluation with a multi-disciplinary team (MDT) to indicate the possible position of the epileptic focus, the SEEG electrodes were implanted. Under local anaesthesia, the patient's head was fixed using a stereotactic frame (Elekta Inc, Stockholm, Sweden), and then CT and contrast-enhanced MRI scans were performed. After the X, Y, Z coordinates were precisely determined, the electrodes were stereotactically inserted to a depth indicated by the calculated values. The exact number and configuration of electrodes varied among the patients and were individually tailored based on the conclusions of the MDT. The electrode locations of one representative patient are listed in Supplementary Table 2. After implantation, all patients underwent CT to confirm the localization of each contact before SEEG monitoring and to ensure there was no haemorrhage in the brain.

1.2. SEEG recording

Intracranial SEEG data were recorded for 72–264 h (3–11 days), and a minimum of 3 seizures were recorded in each patient. A positive SEEG result indicated that there was sufficient evidence to define the seizure-onset zone. In this study, evidence included the first clear SEEG change, such as low-voltage fast activity in the beta and gamma bands, or recruitment and a periodic fast discharge of spikes that occurred before the clinical onset of the seizure[2, 3]. Interictal epileptiform discharges were also considered to be relevant when they consisted of spikes, poly-spikes, spike-and-wave, or poly spike-and-wave complexes. The location and extent of resection were discussed and recommended during a second MDT meeting after reviewing results of the SEEG recordings.

1.3. Surgical procedure and outcome assessment

Guided by the positive SEEG results, resection was performed at the same time the intracranial electrodes were removed. For the temporal lobe epilepsy patients, one of three neurosurgeons, experienced in epilepsy surgery, resected a maximum of 6.0 to 6.5 cm of the anterior lateral non-dominant temporal lobe or 4.5 to 4.5 cm of the dominant temporal lobe. The mesial resection included the amygdala and, at a minimum, the anterior 1.0 to 3.0 cm of the hippocampus (most commonly, 4.0 cm). All surgically-treated patients were assessed postoperatively by a neurosurgeon and a neurologist. Seizure outcomes were classified according to the classification proposed by Engel et al.[4]

2. Data processing

2.1. Theory

Calibrated fMRI was used in our study to model CMR_{O2}. In this model,[5, 6] the maximum baseline BOLD signal change M is described by:

$$M = TE \cdot A \cdot CBV \cdot [dHb]^{\beta} \tag{S1}$$

where TE is echo time, A is the proportionality constant of sample and experiment conditions, [dHb] is the concentration of deoxyhaemoglobin, β is the constant of susceptibility depending on the blood in vessels, and CBV is cerebral blood volume. R_2', the relaxation time that consists only of magnetic spins that can be refocused, can be related to [dHb] as deoxyhaemoglobin is paramagnetic as[7, 8]:

$$R_2' = A \cdot CBV \cdot [dHb]^{\beta} \tag{S2}$$

CBF can be related to CBV using Grubb's constant α, according to[9]:

$$CBV = CBF^{\alpha} \tag{S3}$$

Thus, the [dHb] model as proposed by Hoge et al. is given by:

$$[dHb]^{\beta} = \frac{CMR_{O2}}{4 \cdot CBF} \tag{S4}$$

with β as a physical parameter they describe.[6] Combining equations S1–S4, we can get the equation S5 for absolute CMR_{O2}:

$$CMR_{O2} = 4 \cdot CBF \cdot \left(\frac{R_2'}{A \cdot CBF^{\alpha}} \right)^{\frac{1}{\beta}} \tag{S5}$$

While the parameters α and β are commonly used in calibrated fMRI studies, the parameter A is not commonly used and thus relative measurement is used here to avoid its use. Therefore, CMR_{O2} relative to the mean of grey matter can be calculated as:

$$\text{relative } CMR_{O2} = \frac{CMR_{O2,voxel}}{CMR_{O2,mean}} = \frac{4 \cdot CBF_{voxel} \cdot \left(\frac{R_{2,voxel}'}{CBF_{voxel}^{\alpha}} \right)^{\frac{1}{\beta}}}{4 \cdot CBF_{mean} \cdot \left(\frac{R_{2,mean}'}{CBF_{mean}^{\alpha}} \right)^{\frac{1}{\beta}}} \tag{S6}$$

Since the lower part of equation S6 can be treated as a constant for a given subject, and $rCMR_{O2}$ will eventually be converted into *z*-scores, we can remove the lower part of equation S6 and simplify it as Equation 2 in the main text:

$$rCMR_{O2} = 4 \cdot CBF \cdot \left(\frac{R_2^{'}}{CBF^{\alpha}}\right)^{\frac{1}{\beta}} \quad (2)$$

2.2. Image pre-processing

All data were converted into NIfTI format first with SPM12 (Wellcome Centre for Human Neuroimaging, UK). After reoriented to LPS orientation and resampled to 1 mm using BioImage Suite Web (v1.0.0, Yale University School of Medicine, USA) with MATLAB (2020b, The MathWorks, Inc., USA) commands, the images were segmented into white matter, grey matter, cerebrospinal fluid (CSF), and other components using probability maps in SPM12. Then, white matter, grey matter, and CSF images were combined to generate a whole-brain image without the skull and surrounding tissue via MATLAB. All images were aligned to T1-weighted images (linear registration) and then were registered onto the 1-mm MNI template (non-linear registration) using BioImage Suite Web within MATLAB command. Then, the images were smoothed with a Gaussian kernel of 2 mm. All negative values and missing data were set as “not a number” (nan) and our MATLAB scripts ignored them in calculation. The majority of negative CBF values can be attributed to lower blood flow and prolonged arterial transit time of white matter,[10, 11] which was not used in our study.

2.3. Image analysis

For image pre-processing, all images were in MNI 1 mm space. We calculated $R_2^{'}$ maps from T_2 and T_2^* maps. Then, relative CMR_{O2} maps were calculated with CBF and $R_2^{'}$. From the relative CMR_{O2} and SUV_{glc} maps, we calculated relative OGI maps. Then, *z*-score maps of relative CMR_{O2} and relative OGI were calculated as described in Equation 4 in the main text. After that, we compared the mean value of left side and the right side (or affected and contralateral side) in different brain regions using pair-sample *t*-tests in the healthy controls group and the patients group. MATLAB scripts are available from the corresponding author, upon reasonable request.

References

1. Hyder F, Herman P, Bailey CJ, Møller A, Globinsky R, Fulbright RK, et al. Uniform distributions of glucose oxidation and oxygen extraction in gray matter of normal human brain: No evidence of regional differences of aerobic glycolysis. Journal of Cerebral Blood Flow & Metabolism. 2016;36(5):903-16.
2. Ferrari-Marinho T, Perucca P, Dubeau F, Gotman J. Intracranial EEG seizure onset-patterns correlate with high-frequency oscillations in patients with drug-resistant epilepsy. Epilepsy Res. 2016;127:200-6.
3. Lee SA, Spencer DD, Spencer SS. Intracranial EEG seizure-onset patterns in neocortical epilepsy. Epilepsia. 2000;41(3):297-307.
4. Engel JJ. Outcome with respect to epileptic seizures. Surgical Treatment of the Epilepsies. New York: Raven Press; 1993. p. 609-21.
5. Davis TL, Kwong KK, Weisskoff RM, Rosen BR. Calibrated functional MRI: mapping the dynamics of oxidative metabolism. Proc Natl Acad Sci U S A. 1998;95(4):1834-9.
6. Hoge RD, Atkinson J, Gill B, Crelier GR, Marrett S, Pike GB. Investigation of BOLD signal dependence on cerebral blood flow and oxygen consumption: The deoxyhemoglobin dilution model. Magnetic Resonance in Medicine. 1999;42(5):849-63.
7. Boxerman JL, Hamberg LM, Rosen BR, Weisskoff RM. MR contrast due to intravascular magnetic susceptibility perturbations. Magnetic Resonance in Medicine. 1995;34(4):555-66.
8. Shu CY, Sanganahalli BG, Coman D, Herman P, Rothman DL, Hyder F. Quantitative β mapping for calibrated fMRI. NeuroImage. 2016;126:219-28.
9. Grubb RL, Jr., Raichle ME, Eichling JO, Ter-Pogossian MM. The effects of changes in PaCO2 on cerebral blood volume, blood flow, and vascular mean transit time. Stroke. 1974;5(5):630-9.
10. Alsop DC, Detre JA, Golay X, Gunther M, Hendrikse J, Hernandez-Garcia L, et al. Recommended implementation of arterial spin-labeled perfusion MRI for clinical applications: A consensus of the ISMRM perfusion study group and the European consortium for ASL in dementia. Magn Reson Med. 2015;73(1):102-16.
11. van Gelderen P, de Zwart JA, Duyn JH. Pittfalls of MRI measurement of white matter perfusion based on arterial spin labeling. Magnetic Resonance in Medicine. 2008;59(4):788-95.
